# The role and mechanism of compressive stress in tumor

**DOI:** 10.3389/fonc.2024.1459313

**Published:** 2024-09-16

**Authors:** Min Tan, Bingqi Song, Xinbin Zhao, Jing Du

**Affiliations:** ^1^ Key Laboratory of Biomechanics and Mechanobiology, Ministry of Education, Beijing Advanced Innovation Center for Biomedical Engineering, School of Biological Science and Medical Engineering, Beihang University, Beijing, China; ^2^ Beijing Advanced Innovation Center for Biomedical Engineering, School of Engineering Medicine, Beihang University, Beijing, China

**Keywords:** compressive stress, metastasis, mechanical force, cell extrusion, tumor microenvironment

## Abstract

Recent research has revealed the important role of mechanical forces in the initiation and progression of tumors. The interplay between mechanical and biochemical cues affects the function and behavior of tumor cells during the development of solid tumors, especially their metastatic potential. The compression force generated by excessive cell proliferation and the tumor microenvironment widely regulates the progression of solid tumor disease. Tumor cells can sense alterations in compressive stress through diverse mechanosensitive components and adapt their mechanical characteristics accordingly to adapt to environmental changes. Here, we summarize the current role of compressive stress in regulating tumor behavior and its biophysical mechanism from the mechanobiological direction.

## 1 Introduction

Tumor disease, characterized by the uncontrolled growth and proliferation of abnormal cells as a complex medical condition, can be classified as benign or malignant. Benign tumors exhibit slow growth, do not metastasize, and can often be managed through surgery and other medical interventions. In contrast, malignant tumors are invasive, have metastatic potential, and pose a lethal threat to patient survival. Therefore, early diagnosis and therapy of malignant tumors are crucial for enhancing recovery prospects and improving overall health outcomes. The formation and progression of tumors are complex processes driven by various factors. A primary cause is the gradual accumulation of mutations in previously healthy cells. These mutations, induced by genetic, environmental, dietary, and lifestyle factors, can lead to abnormal cellular behavior (1). Over time, such aberrant cells may undergo uncontrolled division and multiplication, resulting in a mass of tumor cells that can invade and damage surrounding tissues and organs.

Tumor tissue density and cellular morphology changes have long been pivotal diagnostic features for pathologists (2). The components of the surrounding extracellular matrix (ECM) continuously change, and its structure undergoes constant remodeling. This ultimately results in an overall increase in ECM stiffness (3–5). In addition, the compressive stress experienced by the tumor cells rises due to the sustained increase of tumor volume in the limited space (6, 7). Thus, the solid stress generated within tumors due to cell growth and surrounding pressures has become a new area of mechanical pathology, garnering widespread attention (6, 8). Solid stress is the force applied or transmitted through the elastic solid phase of the tissue, and can generate tensile stress and compressive stress. The total solid stress is compressive in all directions within the tumor and radially at the interface with normal tissue, while it is tensile in the circumferential direction at the interface with normal tissue (9). High compressive stress can enhance the invasiveness and metastatic potential of tumor cells, thereby increasing the malignancy of cancer. Monitoring changes in tumor tissue stiffness and internal stress is helpful to identify the early malignant features (8, 10) and make targeted therapeutic strategies. In summary, in-depth research about solid stress and ECM remodeling in tumors provides critical insights for understanding tumor biophysics and improving clinical treatment approaches.

The mechanical microenvironment of tumor tissues is significantly altered, with varying mechanical characteristics across different regions (11). Variations in mechanical forces not only influence tumor cell growth and spread but also impact therapeutic efficacy. Tumor cells constantly interact with surrounding cells and stroma in a dynamic microenvironment, activating signaling pathways that promote tumor growth and metastasis (12). Upon acquiring migratory and invasive capabilities, tumor cells may leave the primary tumor tissue, infiltrate the surrounding ECM, or migrate along the blood or lymphatic vessels to a new environment where they continue to proliferate. These infiltrating cells, though relieved from the compressive forces of the primary tumor microenvironment (TME), encounter fluid shear forces from blood flow and mechanical stress from blood vessel constriction (3). Biomechanical forces such as solid stress, matrix mechanics, interstitial pressure, and fluid shear forces modulate the TME and influence cellular behavior (6).

Apart from genetic mutations, alterations in the mechanobiology of TME significantly contribute to cancer progression. Compression forces are observed at various stages of tumor growth, invasion, and metastasis (13). The overcrowding caused by tumor cell proliferation generates substantial pressure on adjacent normal epithelial cells, leading to their death. The ability of mutated cells to adapt to compressive stress can promote hyperplastic growth and potentially lead to malignancy. Cells detect compressive stress through cytoskeletal components, adhesive molecules, and mechanosensitive proteins on their membranes. In response to mechanical stimuli, cells initiate biochemical signaling pathways that enable them to adjust their behavior, thus fostering tumorigenesis. Compressive stress can transform tumor cells from a non-invasive to an invasive phenotype, thereby enhancing tumor growth and invasion (14). Compression stress can also change the shape of blood vessels, reduce the effect of drug therapy, and increase drug resistance in the treatment of tumors (15). Understanding the impact of compression forces on tumor cells may provide valuable insights for developing novel therapeutic strategies.

This review will primarily focus on how tumor cells perceive compressive stress and the associated mechanical signaling pathways. We will also explore the impact of compressive stress on various aspects of tumor disease. Furthermore, we summarize the regulatory mechanisms of cellular crowding that promote cell extrusion, with the aim of providing new perspectives for studying the role of compression in tumor infiltration.

## 2 Mechanisms of increased compressive stress in tumors

**Figure 1:**
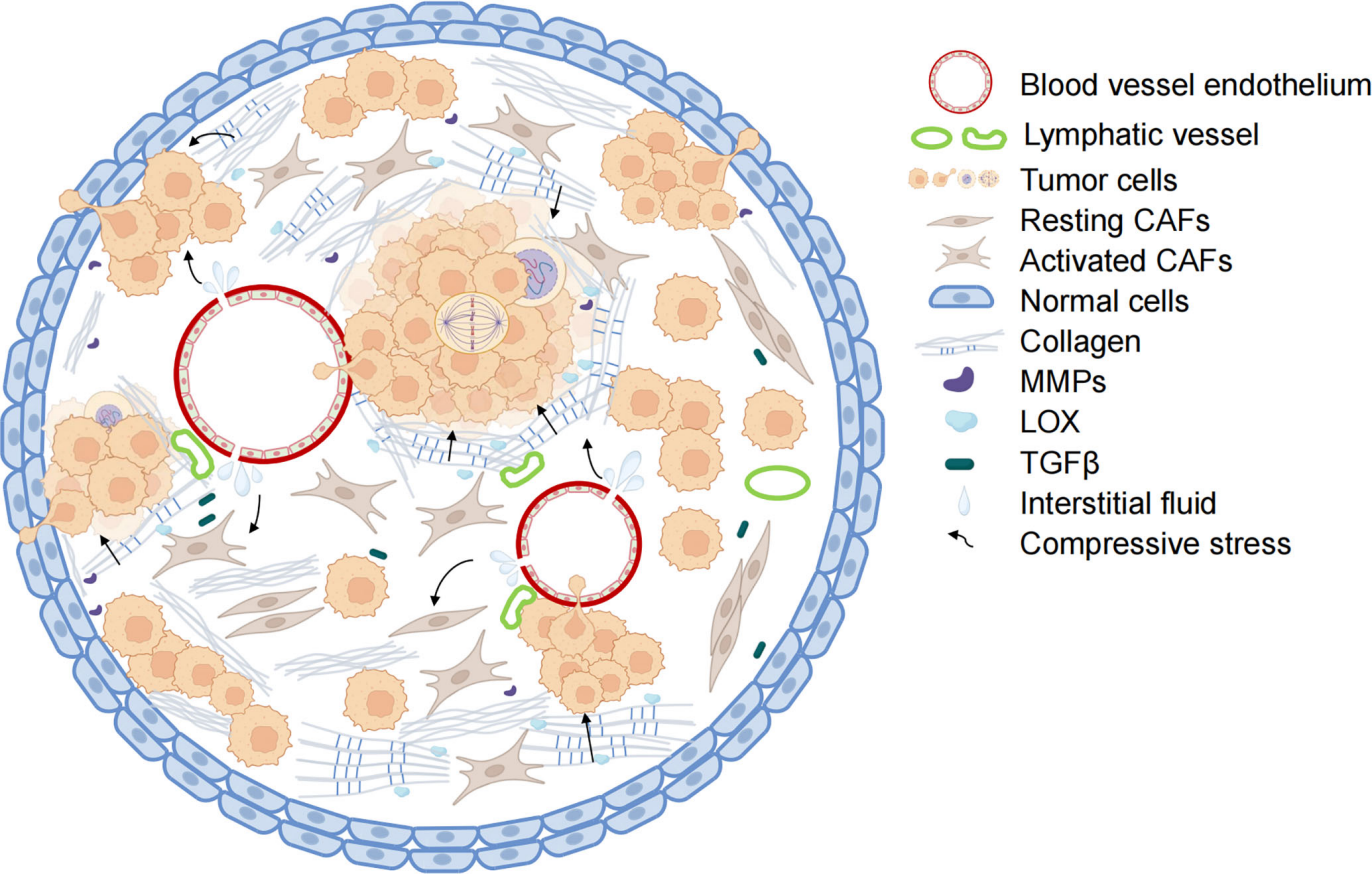
Mechanisms of increased compressive stress in tumors. The combination of blood vessel leakage and pressure on the non-functioning lymphatic vessels leads to the accumulation of tumor tissue fluid and increased interstitial fluid pressure within the tumor. With collagen deposition and crosslinking, the hardness of the tumor extracellular matrix increases. Along with the abnormal proliferation of tumor cells, the tumor tissue has limited enlargement. Under the combined action of the three, the internal compressive stress of tumor tissue also increased rapidly. The blood vessels and lymph vessels are further compressed, and the interstitial fluid pressure is increased. Activated CAFs secrete collagen and LOX to enhance ECM hardness further and promote tumor cell metastasis. (Some illustrations were generated with BioRender.com).

Compressive stress is an important form of mechanical stimulation in tumor development, encompassing both solid compressive stress exerted by non-fluid components and interstitial fluid pressure (IFP) resulting from the interaction between fluid components and tumor tissue (16). With the rapid proliferation of tumor cells and the continuous increase of ECM density, the compressive stress acting on tumor tissue rises significantly (17). This constantly increasing compressive stress influences and impacts various stages of tumor development, including genesis, proliferation, and migration (7, 18). The factors contributing to the increase in compressive stress can be summarized as follows: elevated IFP, abnormal ECM remodeling, and significant tumor tissue expansion (**Figure 1**).

### 2.1 The remodeling of ECM

ECM is a non-cellular component of the TME, primarily composed of macromolecules such as collagen, proteoglycan, and glycoprotein. Functionally, compositionally, and spatially, the ECM can be categorized into two main components: the interstitial connective tissue matrix and the basement membrane. The interstitial connective tissue matrix, which is composed of proteoglycans and fibrous proteins, forms a porous three-dimensional network surrounding cells, contributing to tissue hydration and mechanical strength (19). In contrast, the basement membrane serves as a structural anchor between epithelial and connective tissues (20) and is predominantly constituted by a network of laminin and collagen IV. This basement membrane not only provides essential structural support but also plays a crucial role in establishing cell polarity, which is vital for maintaining tissue homeostasis (21).

ECM plays an important role in maintaining normal physiological functions, providing structural support, and regulating the microenvironment within organisms (22). Additionally, it functions as a reservoir of signaling molecules, thereby facilitating intercellular communication (23). Recent research has established a close link between ECM dysregulation and the onset and progression of various diseases. For instance, excessive ECM accumulation in renal tissue contributes to renal fibrosis, resulting in structural alterations and diminished renal function (24). Moreover, elevated levels of cardiac-specific matrix metalloproteinase 1 (MMP1) lead to collagen degradation and a reduction in contractility, subsequently precipitating cardiomyopathy (25). Abnormal ECM remodeling also represents a significant hallmark of cancer and is integral to tumor progression (26, 27).

#### 2.1.1 ECM remodeling during tumor progression

Cancer-associated fibroblasts (CAFs) are one of the most abundant components in TME and play an important role in the formation and reorganization of the ECM (28, 29). Tumor cells secrete a range of factors such as TGFβ, PDGF, and IL-6, which can activate surrounding stromal cells and induce their differentiation into CAFs. Once activated, CAFs produce various matrix proteins such as fibronectin and type I collagen, resulting in a large amount of ECM deposition (30). Tumor cells and CAFs also promote modification of the ECM by ECM-cross-linking enzymes and the arrangement of collagen fibers by secreting matrix cross-linked lysine oxidase (LOX), which further improves the hardness of ECM (31). Stiff ECM enhances mechanical signal transduction between tumor cells and ECM by promoting the activity of integrin, further changing the direction of collagen and fibronectin in ECM, which promotes tumor growth and metastasis (32).

Matrix metalloproteinases (MMPs), overexpressed by many malignant tumors, are one of the key enzymes of extracellular matrix remodeling. CAFs and tumor cells can secrete and activate matrix metalloproteinases (MMPs) to promote ECM degradation, thus contributing to tumor cell migration (20). MMP-2 and MMP-9 can degrade type IV collagen, the primary component of the basement membrane, and facilitate the metastatic spread of tumor cells (33). Conversely, increased expression of type IV collagen can enhance cell survival. For example, increased expression of type IV collagen in liver tissue will provide a more suitable “soil” for lung cancer cells to be transferred to the liver, which is conducive to the colonization of lung cancer cells in the liver (34).

It is important to note that ECM remodeling is a complex process influenced by many factors. For example, Ras-mutated cells increase actomyosin contractility through the Rho/ROCK pathway (35), and high actomyosin contractility can induce cross-linking and hardening of tumor cells near the stroma (19). ECM remodeling can also act as a feedback signal to affect tumor cell behaviors such as growth and migration, interfere with TME homeostasis, and thus induce further remodeling of ECM (36).

#### 2.1.2 ECM remodeling enhancing compressive stress

The accumulation and compaction of ECM can increase the compression force between tumor cells and adjacent healthy tissue, resulting in elevated solid stress within the tumor and at the interface with healthy tissue (37). This effect parallels the impact of tumor volume expansion. ECM components, such as hyaluronic acid (HA), which can absorb water and swell, contribute to mechanical forces within the tumor tissue, manifesting as solid pressure rather than fluid pressure (38). The remodeling of ECM necessitates the participation of actomyosin and the cytoskeleton (36). When CAFs and tumor cells undergo migration within the TME or engage in tissue repair processes, they activate the cytoskeleton to generate contractile forces on both cells and ECM components, thus generating additional mechanical forces in some specific regions of the tumor (39). The bidirectional between ECM and cells is bidirectional, as alterations in ECM biomechanics can directly induce cytoskeletal reorganization and actin protrusion formation (40, 41), leading to modifications in membrane tension (27).

Furthermore, the harder and denser ECM can compress the surrounding blood vessels and lymphatic vessels, leading to blood leakage and damage to the lymphatic system. Increased IFP caused by compression of blood vessels and lymphatic vessels creates a local hypoxia environment that hinders drug delivery (42, 43). Animal experiments have proved that reducing ECM density and stiffness can effectively improve IFP (44). A transgenic model of preclinical pancreatic ductal adenocarcinomas revealed that after enzymatic degradation of HA, the compressive stress was reduced, the delivery efficiency of the anti-tumor drug Gemcitabine was increased, and the survival rate of mice was significantly improved (44, 45).

### 2.2 The growth of the tumor tissue

Tumor cells can circumvent growth inhibition and continue to proliferate (46), providing a foundation for the uncontrolled expansion of tumor tissue. During the proliferation process, tumor cells displace the existing viscoelastic structures within and surrounding the tumor, thereby generating additional solid stress (6). For tumor cells, the continuous expansion into the surrounding tissues also causes coordinated displacement of surrounding normal tissues, that is, when the tumor pushes against the surrounding tissues, the surrounding tissues will also restrict the activities of the tumor. This restriction transfers a large amount of mechanical force to the tumor tissues through intercellular adhesion (47). In addition, the huge tumor tissues can also compress blood vessels and lymphatics along with the ECM, resulting in the extravasation of tissue fluid and higher IFP (6, 48). It will further increase the compressive stress.

### 2.3 Increased interstitial fluid pressure

Increased IFP caused by fluid accumulation is important in increasing compressive stress within the tumor. Numerous studies have shown that the surrounding blood vessels undergo vascular remodeling (49) and abnormal changes in morphological structure during the development of tumors (50). For example, blood vessels in tumor tissue are mostly saccular, tortuous, and disorganized (51), and show higher permeability than normal blood vessels (52). The high permeability of blood vessels causes an increased leakage of interstitial fluid, leading to a buildup of fluid in the tumor tissue, further elevating the tissue pressure (6, 48). Compared with the IFP of normal tissue which was close to 0 mmHg, the IFP of most tumor tissues was between 5-40 mmHg (53). The IFP difference between tumor tissue and surrounding tissue will form a steep IFP gradient at the tumor boundary, promoting growth factors and tumor cells to enter surrounding normal tissue and lymphatic vessels, and promoting tumor migration and invasion (54). On the other hand, the inflow of growth factors will provide more nutrients for tumor tissues and further lead to the volume expansion of tumor tissues. Therefore, elevated IFP is frequently utilized as a diagnostic marker for malignancies of the breast, head, and neck (55, 56) and is also assessed as a prognostic indicator in certain clinical studies (57).

## 3 Effect of compressive stress on tumor progression

Approximately 90% of cancers originate in epithelial cells. During the early stage of tumorigenesis, abnormal cell proliferation occurs in the epithelial tissue, forming hyperplastic lesions known as intraepithelial neoplasia. This results in progressive overcrowding of epithelial cells while remaining non-invasive at this stage, making it a good window for intervention. In conventional cell culture models, it has been observed that cells produced contact inhibition, and lateral pressure inhibited cell proliferation when cell confluence reached stability (58). Similarly, in a closed and confined microenvironment, the growth cycle of tumor cells is inhibited (59). This is a body’s self-protection mechanism at the early stage of cancer. However, tumor cells may reduce their sensitivity to overcrowding by regulating the expression of mechanosensitive proteins, thereby gaining a competitive advantage. This reduces the activation of the apoptosis signal, allowing extruded tumor cells to continue to grow and proliferate. Previous *in vitro* studies have shown that strong compression forces can inhibit tumor growth (60) and even induce the collapse of blood vessels or lymphatic vessels in tumors in the body (15, 61), thereby reducing the supply of oxygen and nutrients, and leading to the formation of necrotic tissue inside the tumor. However, compression also reduces blood perfusion and affects drug delivery, which can interfere with tumor treatment (62, 63). Even in the later stages of tumor development, compression force can facilitate tumor cell proliferation and migration (64).

### 3.1 The promotion of primary tumor development by compressive stress

Research on cellular competition has gained attention in recent years, particularly about how cells with mutations gain a survival advantage and destroy healthy cells during competition. Tumor cells, which can proliferate rapidly, compete with surrounding cells for nutrients and space. However, healthy epithelial cells can recognize some of the mutated cells, squeeze out them and remove abnormalities to maintain tissue homeostasis. This process is called epithelial defense against cancer (EDAC) and eliminates sub-optimal cells in the tissue (65, 66). This is a process of cellular competition in which sub-optimal cells in the tissue are eliminated (67).

Despite the tumor suppressive effect of EDAC, tumors often appear in epithelial tissues, which may be due to the mechanical competitive advantage of tumor cells. Different cell types of the human body may have different sensitivity to compression forces, and those cells sensitive to compression forces may trigger apoptosis signaling pathways at lower compression forces, leading to apoptosis (68). Compared to rapidly growing tumor cells, the surrounding slow-growing cells will die under the compressive force (69). In the cell mass, highly YAP expressed cells showed stronger competitiveness and faster proliferation and expansion (70, 71), suggesting that cells with high YAP levels are better able to withstand the mechanical stresses imposed by their environment. Studies have shown that hyperactivation of YAP in peritumoral hepatocytes triggered regression of primary liver tumors and melanoma-derived liver metastases (72). The reprogramming of normal cells into tumor-initiating cells by Ras oncogenes requires increased force transfer between cells and the ECM. In this process, YAP/TAZ is required to regulate transduction mechanical signals and adjust the initial carcinogenic potential of tumor cells (73).

The mechanical properties profoundly affect cell fate, such that cells with higher tension (harder cells) tend to be devoured by cells with lower tension (softer cells) (74). In a heterogeneous population, tumor cells with high deformability preferentially engulf and outpace neighboring cells with low deformability. K-Ras^G12D^ mutant cells are recognized by normal cells through increased expression of membrane receptor EPHA2, and the mutant is mechanically extruded from tissues due to reduced adhesion and competitiveness (75). On the contrary, if EPHA2 is knocked out, the K-Ras^G12D^ mutant cells will rapidly multiply to form clones. In conclusion, in the early stage of tumor development, mutated cells may reverse the effect of compressive force after gaining a competitive advantage in mechanosensory conduction.

### 3.2 Cell extrusion

Normal epithelial tissue can maintain the homeostasis of epithelial cell numbers by sensing cell density. When cell density is too high, epithelial cells can recognize overcrowding and push out cells from the crowded area towards the lumen from the apical end. The displaced cells then undergo apoptosis (76). Previous studies have shown that cells about to be extruded release sphingosine-1-phosphate (S1P), which activates actomyosin contraction in surrounding cells through the S1P2 receptor (77). Actin contraction rings form around the extruded cells and extrude them from the epithelial lamellar layer. Basal adhesion loss triggers cell death by activating the apoptotic signaling pathway in extruded cells.

Cell extrusion from the apical surface of the epithelium are typically associated with tissue remodeling or the removal of damaged or dead cells. Correspondingly, cell extrusion from the basal surface of the epithelium are involved in maintaining tissue homeostasis and usually occur through mechanisms such as disruption of cell-cell junctions and cell migration. Cells with carcinogenic mutations can activate the survival signaling pathway, change the direction of extrusion, and escape the fate of apoptosis. Basal cell extrusion (BCE) may trigger tumor cells to break through the basement membrane to proliferate and metastasize (78). Cells with K-Ras mutations prefer basal extrusion relative to apical extrusion and can survive and proliferate after extrusion. Degradation of S1P in K-Ras^V12^ mutated cells blocks apical cell extrusion (ACE) and promotes basal extrusion (79). The atypical protein kinase Ci (aPKCi) is an oncogene whose overexpression can also trigger BCE (80). aPKCi drives cell segregation by influencing vinculin localization at the cell-to-cell junction. The microtubule regulatory factor adenomatous polyposis coli (APC) seems to be the main force promoting basal extrusion. Cells lacking APC or expressing carcinogenic APC mutations drive basal extrusion (81). In APC mutant cells, the microtubules are unstable and cannot target APC to the bottom of the cell to control where contraction occurs. As a result of the loss of targeting, the top end shrinks, causing the base to extrude.

The initiation of tumor cell invasion and migration may be partly attributed to BCE. Reduced E-cadherin expression observed in some metastatic tumors decreases intercellular adhesion, facilitating extrusion (82). The transcription factor Snail is highly expressed in cancer tissue and promotes epithelial-mesenchymal (EMT). In contrast to its transcriptional role, Snail enhances apical contraction by regulating E-cadherin and RhoA during EMT, which allows cells to be extruded towards the basal side (83) and aids in tumor cell metastasis. Overexpression of aPKCi regulates vinculin localization at intercellular junctions, promoting BCE. Conversely, down-regulation of aPKCi impedes EMT and limits migration and invasion of breast cancer cells (84). Structural centrosome aberrations induced by ninein-like protein preferentially squeeze epithelial cells out of the basement (85) and induce cell metastasis when mutations and centrosome aberrations occur at the same time.

### 3.3 Cell migration

With the continuous development of tumors, the living space of cells is limited, and the strong compressive stress directly or indirectly promotes the process of tumor cell migration. For the entire tumor tissue, the compression force is enough to compress the blood vessels, reducing the oxygen supply to the tumor cells. Hypoxia is a common phenomenon in solid tumors. In contrast to normal cells, hypoxic tumor cells activate several survival pathways as an adaptive response to hypoxic stress. For example, hypoxia induces tumor cells to degrade the extracellular matrix and escape from hypoxic regions to oxygen-rich environments at secondary sites (86, 87). Blood vessel compression and solid stress protect cancer cells from the host immune system, and even when immune cells successfully reach tumor tissue (88), lack of oxygen impairs their killing potential (89).

During the process of tumor metastasis, tumor cells that have acquired the ability to invade and migrate leave the primary tumor, spread to neighboring tissues, or travel to the distal landing via blood vessels through multiple mechanisms. The mechanism by which epithelioid cancer cells transform into mesenchymal cells to acquire the ability to move and spread is still controversial, but it is well-studied and widespread (90, 91). Cell extrusion is inextricably related to EMT transformation of tumor cells, considering that both of them are perhaps the simplest course of action for tumor cells to initiate invasion and metastasis. EMT is characterized by changes in cell morphology, as well as intercellular and cell-matrix adhesion (91). Compressive stress induces glycolysis of human breast CAFs, thereby contributing to the expression of EMT and angiogenesis related genes in breast cancer cells (92). During EMT, the loss of E-cadherin allows tumor cells to separate from surrounding cells and colonize new living spaces, which is often observed in metastatic tumors. The weakening of adhesion is conducive to cell extrusion. Hypoxia can promote tumor invasion and metastasis by activating the hypoxia-inducible factor (HIF) signal and regulating various transcription factors related to the EMT process (93, 94).

For individual tumor cells, compression and low adhesion induce rapid amoebic migration of mesenchymal cells due to cell space limitations (95). This amoeboid-like cell movement depends on the force generated by cortical actin contractions. Compressive stress causes cells to adopt an amoeboid mode to achieve higher mobility. Hosseini et al. found that tumor cells adjust their cell division mechanisms through EMT to adapt to crowded environments. Actually, overcrowding still impedes cell division, leading cells to escape and migrate away from the confined space (96). In short, the crowded TME caused by abnormal proliferation affects various life activities of the tumor, thus promoting the invasion and metastasis of tumor cells.

### 3.4 Metabolic alterations

Metabolic reprogramming is a characteristic of cancer (97). Research in the early 1920s found that the rate of lactic acid fermentation of tumor cells was very high regardless of anaerobic or aerobic conditions, which indicated that glycolysis replaced oxidative phosphorylation as the main feature of tumor cell metabolism^122,1^. There has been growing evidence of a relationship between mechanical signals and tumor cell metabolism. Compressive stress can be involved in the regulation of a variety of metabolism-related signaling pathways (98, 99). Compressive stress alters cellular matrix adhesion, promotes cytoskeletal remodeling, and regulates actomyosin contractility. Several studies have found that the cytoskeleton further regulates cellular metabolism (99–101). When actin contractility increases, it consumes a lot of energy and improves the level of glycolysis adaptively (99). RhoA/ROCK signaling may couple or coordinate actomyosin dynamics with mitochondrial dynamics for optimal actomyosin function, resulting in protruding and migration behavior (102). YAP/TAZ is a classical mechanosensitive signaling pathway that has been shown to bridge mechanical forces with the metabolic reprogramming of tumor cells (73, 103, 104). Stiff matrix stiffness increases ATP production from OXPHOS through YAP mechanical transduction and promotes tumor invasion (105). Activation of the YAP/TAZ signaling pathway will increase glycolysis and glutamine metabolism, thereby coordinating non-essential amino acid flux within the tumor niche and promoting tumor cell proliferation (106).

Adenosine 5 ‘-monophosphate-activated protein kinase (AMPK) plays a key role in maintaining energy homeostasis and is a “guardian of metabolic homeostasis” (107, 108). Mechanical forces can activate AMPK via E-cadherin, driving glucose uptake and ATP production (101). Increased ECM stiffness also induces AMPK-dependent downregulation of lipid synthesis in pancreatic stellate cells, which promotes the progression of pancreatic adenocarcinoma (109). In addition, to adapt to the anoxic environment caused by the increase of compressive stress, tumor cells will up-regulate the HIF to improve the level of glycolysis (110). At the same time cells stimulate the biosynthesis pathway of hexosamine to ensure their survival (111). The tumor can adjust its energy distribution according to the size of the space, reducing energy costs and guiding its path selection as it migrates through confined spaces (112).

It is essential to note that the effect between mechanical stimulation and tumor cell metabolism is mutual (40, 113). High ECM stiffness up-regulates the expression of SLC1A3 transporter and glutamic amidase 1 in CAFs, promoting glutamate uptake and glutathione synthesis, and further promoting ECM remodeling (106). When Ras^V12^ mutant cells are surrounded by normal cells, decreased mitochondrial membrane potential leads to dysfunction, increased glucose uptake, and enhanced glycolysis (114). The phenotypic changes of glycolytic metabolism make Ras^V12^ mutant cells to be squeezed, which is not conducive to tumor cell survival (114). However, in the stage of rapid proliferation of tumor cells, glycolytic metabolism becomes critical, suggesting that changes in metabolic phenotype may alter EDAC and affect cancer resistance (115).

## 4 Mechanical force-sensitive element

**Figure 2:**
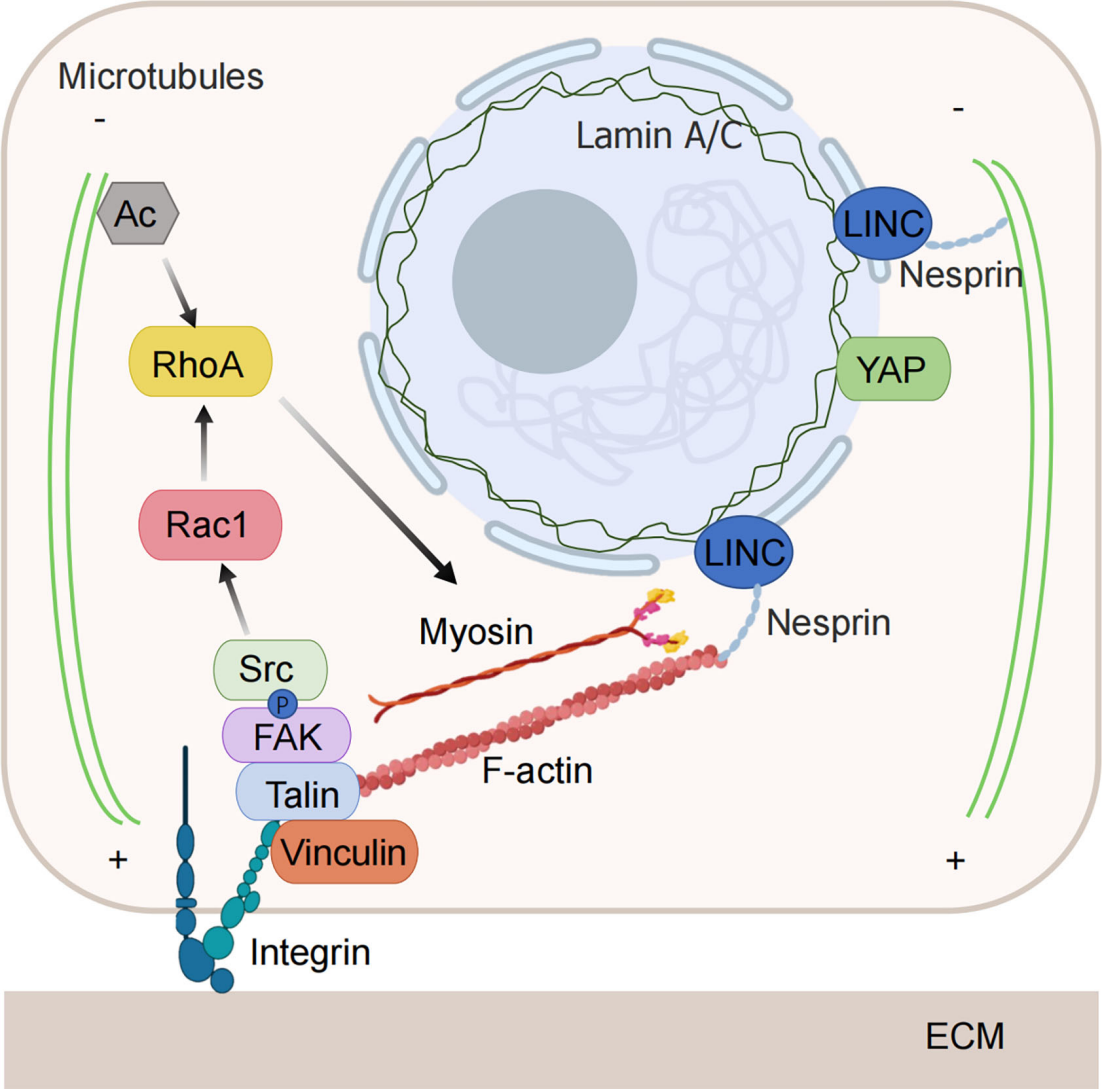
Mechanical force-sensitive element. Tumor cells have the ability to detect changes in the level of compressive stress surrounding them and can alter their behavior to adapt to these stressful conditions. The increased compressive stress is sensed by mechanical sensors within the cell and transmitted to the interior through various mechanosensitive pathways, converting it into biochemical signals that govern cellular functions. When subjected to compressive stress, both the nuclear membrane and the nucleoskeleton can detect nuclear deformation, initiating downstream signals. The cytoskeleton is also capable of directly sensing and responding to compressive stress through structural reorganization. Integrins are activated and regulate the cytoskeleton via downstream signaling pathways under the influence of compressive stress. (Some illustrations were generated with BioRender.com).

Tumor cells can sense changes in the level of compressive stress around them and regulate cell behavior to adapt to the stressful environment. The increased compressive stress can be sensed by mechanical sensors in the cell and transmitted to the cell interior through a variety of mechanosensitive pathways, transforming it into biochemical signals that regulate cellular activities (**Figure 2**).

### 4.1 Nucleus

Recent studies suggest that the nucleus plays a crucial role in how the cell senses compressive stress, making it an increasingly important focus in cancer research. Compared to the flexible cell membrane and cytoskeleton, which can undergo significant deformation, the nucleus is less flexible and more sensitive to mechanical forces and deformation (116).

When tumor cells migrate through compressed spaces, the cells undergo severe compression and change their shape through cytoskeletal remodeling as they pass through narrow tissue pores. The stiffer nucleus can act as a ‘ruler’ to gauge the confined space and respond by adjusting the cytoskeleton to fit the environment (117, 118). During this migration, the nucleus experiences significant deformation, which may lead to the rupture of the nuclear membrane and cause DNA damage. However, the damage to the nuclear membrane and DNA is repaired to prevent cell death (119). Repeated loss of nuclear membrane integrity during extrusion is an important driver of the invasive phenotype of tumor cells (120).

The nuclear membrane not only plays a role in protecting chromatin, regulates material and information exchange inside and outside the nucleus but also participates in mechanical force transmission and response. Both compressed and deformed nuclei and stretched nuclear membranes reduce the resistance of molecular transport of nuclear pores and affect transcriptional regulation (121, 122). Lamin A/C is a major structural protein of the nuclear membrane, which forms a skeleton structure supporting the inner side of the nuclear envelope, participates in the protection of the genome, and is mechanically sensitive. Silencing lamin A/C to reduce nuclear stiffness increases nuclear compression and YAP nuclear localization (123). Additionally, extrusion pressure can directly stretch the nuclear membrane or nuclear pore, triggering downstream signal transduction independently of the cytoskeleton and other mechanosensitive pathways.

When mechanical forces are applied to the cytoskeleton, they can also be transmitted to the nucleus via the linker of the nucleoskeleton and cytoskeleton (LINC) complex. The nesprin protein in the LINC complex binds to actomyosin and microtubules on the cytoplasmic side and binds to lamins and inner nuclear membrane proteins on the inner side of the nucleus (124). Through the function of the LINC complex, the nucleus can respond to mechanical forces by altering nuclear structure, chromosome recombination, and gene expression (125). Knocking out NUP210 inhibits the LINC complex from linking to chromatin, thereby disrupting the mechanosensitivity of the nucleus and resulting in decreased tumor cell metastasis (126). In contrast to this passive influence mechanism, chromatin itself also mediates mechanical sensitivity, protecting the genome from stress by driving nuclear malacia (127).

### 4.2 Cytoskeleton

Many studies have found that integrins, G protein-coupled receptors, transient receptor potential (TRP) ion channels, piezoelectric channels, and YAP/TAZ mechanical sensors can detect mechanical forces and regulate cytoskeletal remodeling, thereby guiding cell response to mechanical changes. The cytoskeleton also directly senses and transmits mechanical forces (41, 128).

The cytoskeleton is mainly composed of actin filaments, microtubules, and intermediate filaments. When compressive stress is applied to cancer cells, some binding proteins on the actin filament act as mechanical sensors that sense the mechanical forces, responding to them by dynamically adjusting the assembly and disassembly of F-actin and G-actin (99, 129). The actin filament itself can also sense the change in tension and adjust the affinity with the filament to determine the length of the actin filament (130). Therefore, compressive stress can change cell tension and affect cytoskeletal remodeling. As a mechanical sensor, actin filaments bind to the motor protein myosin II to generate contractile forces that push the plasma membrane forward, which plays an important role in tumor cell migration (131). The compressive stress applied to cells and tissues increases regulatory myosin phosphorylation, actomyosin contractility and tension via ROCK (132). In the process of tumor compressive stress increase, tumor cells can sense the hardening of the extracellular matrix through the change of cytoskeleton contractile force, rapidly adjust the structure of the actin network to achieve cell migration.

This compressive stress from the tumor microenvironment regulates the hardening of the cytoskeleton, and the microtubules (MTs) act as compression-bearing elements. As an important component of cilia and flagella, MTs play an important role in material transport and mitosis of cells. Microtubules respond to compressive stress in living cells and find that microtubules become distorted, less dynamic and more stable (133). This process seems to help tumor cells migrate in a crowded environment. Studies have shown that MTs can also regulate the mechanical sensitivity of adhesion plaques and YAP (134). MT acetylation can stimulate RhoA and increase the contractility of myosin (134). The increase of ECM stiffness makes MT glutamylation stable, which is conducive to cancer cell invasion (100). Intermediate filaments are usually assembled around the nucleus and are more stable than microtubules and microfilaments. Due to its stable and elastic properties, it is a key component for tumor cells to sense the direction of mechanical stress (135).

### 4.3 Integrin-FAK

Integrin is a common mechanosensitive transmembrane protein that plays a key role in tumor cell adhesion, migration, and signal transduction (136). Integrins connect the cytoskeleton and extracellular matrix, sensing changes in extracellular matrix stiffness. They consist of α and β subunits that can combine to form different types of integrins (137). The activation of integrins triggers intracellular signaling through the cytoskeleton (138), regulating tumor cell invasion, migration, and other cell behaviors. Proteins inside the cell activate integrins by binding to the integrin-β subunit on the inside of the cell, and can also activate integrins by binding to various ligands outside the cell. After ligands bind to the extracellular domain of integrin, cytoskeletal proteins such as talin and vinculin are recruited to bind to integrin, thus promoting the conformational changes activated by integrin (137). Talin connects to the cytoskeleton, promotes the further aggregation and activation of integrins, and subsequently initiates intracellular signal transduction, activating the FAK and SRC families (139).

Loading compressive force on cells increases the expression of integrin, induces the formation of stress fibers, and enhances the transmission of mechanical force signals in combination with cytoskeleton (140). The compressive stress enhances integrin affinity maturation and promotes integrin αIIbβ3 adhesive function (141). Forces acting on the extracellular matrix activate integrins by pulling on ligands that bind to the integrins. Integrins transfer these forces from the cell membrane to the actin cytoskeleton by binding to actin-binding adaptor proteins (142). Cross-linking of collagen in the extracellular matrix in breast tumors has been found to lead to sclerosis of the extracellular matrix, and the hardened basement regulates cell contractility and promotes aggregation of integrins to form focal complexes, thereby facilitating downstream signal transduction (138). On the contrary, the rate of integrin recruitment to vinculin is reduced on the soft matrix (143), leading to a weakened connection to the cytoskeleton and consequently maintaining a low conduction level.

In a variety of tumor diseases, the phenomenon of extracellular matrix stiffening has been observed. In addition, it has been revealed that the integrin expression level increases in various tumor diseases, further enhancing the integrin-related signaling pathway (144). When mechanical force signals are present, conformational changes in integrins are stimulated. Activated integrins trigger the phosphorylation of FAK, causing FAK to bind to SRC to form complexes (137). FAK/SRC complex is involved in various signaling pathways and plays an important role in promoting cell adhesion, proliferation, migration, and differentiation (139). Integrins also promote cell survival through several other signaling pathways. For example, phosphatidylinositol 3-kinase (PI3K)/protein kinase B (AKT) pathway/mitogen-activated protein kinase (MAPK)/extracellular regulated kinase (ERK) pathway, etc (136). Cells with carcinogenic mutations may activate these signaling pathways through integrin, escaping their apoptotic fate in a squeezed environment. Actomyosin contractile force is activated by the FAK/Src/ROCK signal, which drives cell sensing substrate rigidity and regulates cell migration (4, 145). The integrin-activated adaptor protein Kindlin recruits paxillin to the nascent adhesion to activate Rho GTPase Rac1 and binds to the actin-polymerized Arp2/3 complex to mediate cell membrane protrusions (146). Integrins also regulate cytoskeletal assembly through talin and KANK connections on microtubules (146).

## 5 Mechanism of overcrowding promoted cell extrusion

Cell extrusion coordinates morphogenesis by removing apoptotic cells and mediates cell competition during tumorigenesis, acting as a key regulator of epithelial homeostasis. In the process of tumor development, the abnormal cell extrusion mode promotes the survival advantage of tumor cells to escape the fate of apoptosis, and then invade and colonize distant organs. This process involves changes in a variety of signaling pathways, and in-depth exploration of the regulatory mechanism of cell extrusion will provide a new strategy for tumor treatment and improve the therapeutic effect on tumors.

### 5.1 Extrusion triggered by intercellular signal transduction

**Figure 3:**
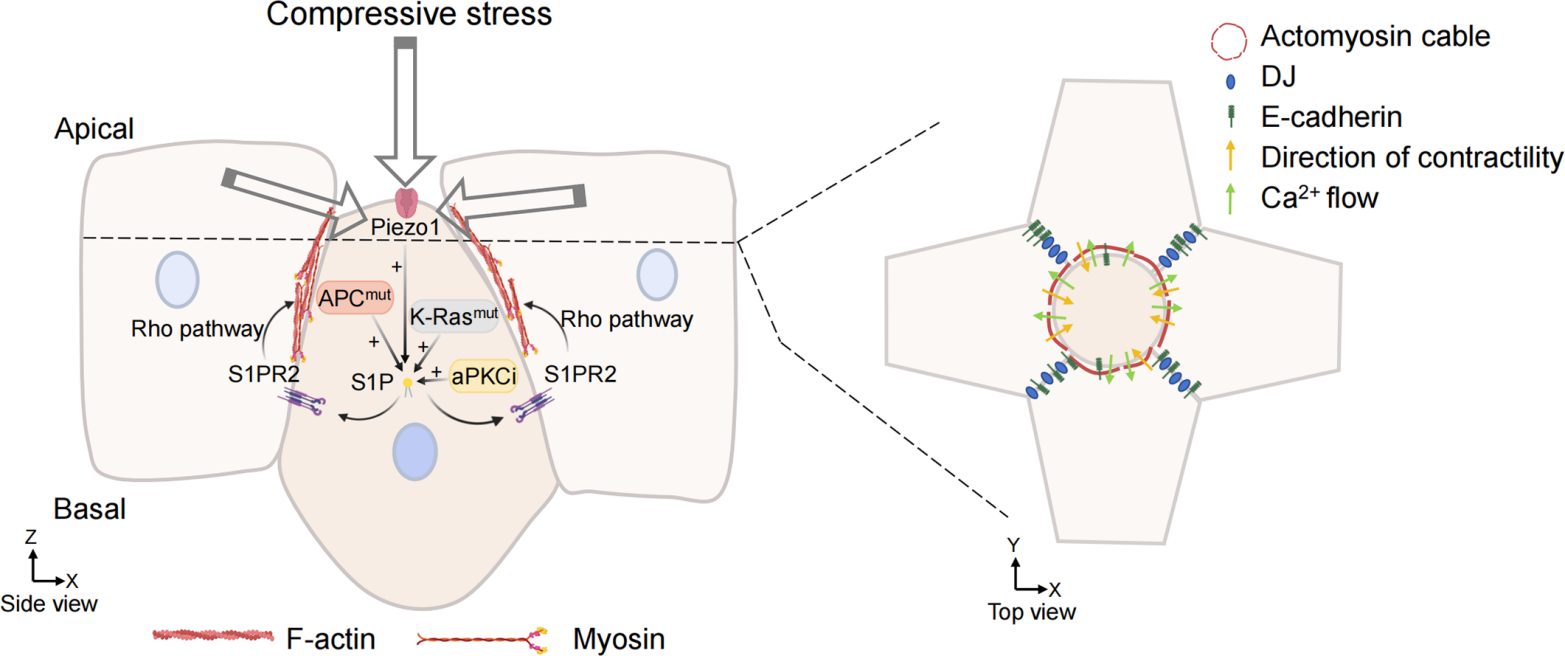
Overcrowding-induced basal cell extrusion. APC, K-Ras mutations, and aPKCi promote tumor cells basal extrusion. Cells destined for extrusion produce sphingosine-1-phosphate (S1P), which binds to the S1P receptor (S1P2) in neighboring cells. S1P2 promotes the formation of actomyosin contraction ring through Rho signal transduction and triggers basal extrusion of cells. When the mechanosensitive ion channel is activated by compressive stress, the calcium ion fluctuation also promotes actin remodeling of surrounding cells and promote cell extrusion. Cell junction, adhesion, and intermediate filaments are also involved in the formation of actomyosin rings and regulate their contractility. (Some illustrations were generated with BioRender.com).

S1P is a signal released by the cell that is about to be extruded to initiate the extrusion process. S1P binds to a type 2 receptor (S1PR2) in neighboring cells to trigger the assembly of intercellular actin rings (**Figure 3**). This communication of signals between cells is a key link in triggering cell extrusion. Blocking the entry of S1P into surrounding cells or inhibiting the formation of S1P can prevent the extrusion of apoptotic cells (77). The mechanically sensitive ion channel Piezo1 may play a role as the upstream of the S1P signal in maintaining epithelial cell homeostasis. Inhibiting or knocking out Piezo1 prevents the death of cells due to overcrowding and leads to the accumulation of cell clumps at sites where epithelial cells are crowded (76).

In recent studies of crowding-induced living cell extrusion, it was found that the activation of the mechanically sensitive ion channel Piezo1 induces living cell extrusion (76, 147), which produces calcium ion fluctuations for signal transduction. Calcium ions are fundamental second messengers that activate multiple pathways to regulate cellular function. The signal transduction generated by calcium ion fluctuations can initiate actin contraction of surrounding cells, forming actin contraction rings and promoting cell extrusion in the center (**Figure 3**). When Ras^V12^ mutant MDCK cells were mixed with normal MDCK cells, Ras^V12^-expressing cells were often squeezed out. It was observed that the level of cytoplasmic calcium was increased in the extruded Ras^V12^ mutant cells, and calcium ion fluctuations were formed to propagate to the distal cells (79). Takeuchi et al. found that the mutated cells destined for extrusion induce calcium ion fluctuations through the mechanosensitive calcium channel TRPC1 (148). TRPC1 is needed for calcium waves and extrusion in this study, and the activation of Piezo1 could do the same. In both zebrafish embryos and mammalian epithelial cells, the phenomenon of calcium ion fluctuation during cell extrusion was found, and the mechanism of calcium ion spread in the cell population was very similar (149), indicating that calcium wave is an evolutionarily conserved and universal mechanism for cell extrusion.

### 5.2 Actin remodeling

The cell compressive force of cell extrusion is primarily driven by myosin ring contraction. Lamellar pseudopodia creeping towards the cell base of adjacent cells also plays an important role in ACE. The extruded cell communicates with surrounding cells, triggering the surrounding cells to form actin filaments around the extruded cell and promoting actin contraction to extrude the cell (150). Myosin II is phosphorylated through activation of the Rho and Rho-associated kinase pathways, resulting in actomyosin contraction (132). Once forming and contracting on the apical side, the actomyosin ring on the surrounding cells promotes cell extrusion on the basal side.

Cell junction, adhesion, and intermediate filaments are also involved in the formation of actomyosin rings and regulate their contractility (**Figure 3**). It was found that desmosome junction (DJ) defects would lead to cell extrusion failure during cell extrusion. Intact DJ ensures mechanical coupling between cells and maintains actin contraction, which is necessary during cell extrusion (151). E-cadherin is necessary for cell extrusion (152, 153). E-cadherin knockdown did not affect the extension of lamellar pseudopodia but interfered with the formation of actin contraction rings. E-cadherin can transfer the mechanical force brought about by the contraction ring, and the only plate pseudopodia cannot compensate for the force of the actin ring and cannot promote cell extrusion.

The soluble lipid S1P is an apoptotic signal that can activate RhoA, while the extruded cells activate RhoA in their neighbors through E-cadherin and myosin-dependent mechanical transduction, ensuring that the surrounding cells form extrusion pressure (153). Other studies have found that the way the cell extrusion mode is different due to the difference in cell packing density. In confluent epithelial cells with low cell density, cell extrusion is primarily driven by a plate-footed crawling mechanism in neighboring non-dead cells. As cell density increases, the formation of actomyosin cables on the cell and their contraction in neighboring cells become the main mechanisms that locally promote cell extrusion (154). In tumor tissue, crowded environments may also rely on these two mechanisms above when they promote cell extrusion.

## 6 Discussion

The influence of compressive force on the growth, development, and maintenance of the human body is far more than the content discussed in this review, and it is involved in the occurrence and development of many diseases. Excessive compression can cause intervertebral disc degeneration, which occurs due to a large number of cell deaths and failing to maintain homeostasis induced by increased compression, ultimately leading to disease (155). Additionally, the compressive stress caused by cell crowding also regulates the growth of intestinal organoids (156). The way compressive stress acts in these diseases may bring implications for the study of tumor diseases.

Reducing ECM deposition through pharmacological intervention may mitigate the effects of compressive stress and inhibit tumor progression. Previous studies demonstrated that saridegib alleviated growth-induced stress by inhibiting CAF proliferation and suppressing collagen production within the extracellular matrix. This approach reduces solid stress, alleviates compressive stress in tumor tissues, restores vascular diameter, and increases the number of functional blood vessels (15). The combination of gemcitabine and all-trans retinoic acid clinically can significantly inhibit cancer cell proliferation, reduce ECM deposition, increase vascular distribution, and enhance anti-tumor efficacy (157). Injection of hyaluronidase by directly degrading the extracellular matrix can reduce capillary pressure within tumor tissues. When combined with liposomal doxorubicin, this approach can enhance therapeutic efficacy (158).

Compressive stress has been identified as a factor in influencing tumor cell behavior, prompting tumor cells to adapt to complicated mechanical environments through mechanical force sensors. However, further research is required to elucidate how tumor cells appropriately respond to varying levels of compressive stress in diverse environments. The relationship between compressive stress and tumor development remains a controversial issue. Therefore, more ingenious and sensitive methods are necessary to characterize the compressive forces sensed by cells, bringing more impartial judgment to future research.

In conclusion, it is necessary to understand the influence of compressive stress on various links of tumor diseases for the research and treatment of tumor diseases. Intervening compression forces in tumor development may present new therapeutic strategies.
